# Bone microarchitecture assessed by High-resolution peripheral quantitative computed tomography in type 1 diabetes: the Diabetes Control and Complications Trial/Epidemiology of Diabetes Interventions and Complications study

**DOI:** 10.1093/jbmrpl/ziaf167

**Published:** 2025-10-22

**Authors:** Mishaela Rubin, Galateia Kazakia, Barbara H Braffett, Naina Sinha Gregory, Ming-Hui Lin, Victoria Trapani, Ian H de Boer, Sanchita Agarwal, Andrew Burghardt, X Edward Guo, Rose Gubitosi-Klug, Ann V Schwartz, For the DCCT/EDIC Research Group, Valerie Arends, Jye-Yu C Backlund, Annette Barnie, Ionut Bebu, Barbara H Braffett, Andrew Burghardt, Ian De Boer, Kaleigh Farrell, Naina Sinha Gregory, Rose Gubitosi-Klug, Galateia Kazakia, David J Kenny, John M Lachin, Ming-Hui Lin, Thomas Link, Mishaela Rubin, Ann V Schwartz, Victoria R Trapani, Amisha Wallia

**Affiliations:** Division of Endocrinology, Columbia University Irving Medical Center, New York, NY 10032, USA; Department of Radiology and Biomedical Imaging, University of California San Francisco, San Francisco, CA 94143, USA; The Biostatistics Center, George Washington University, Bethesda, MD 20817, USA; Division of Endocrinology, Diabetes, and Metabolism, Weill Cornell Medicine, New York, NY 10021, USA; The Biostatistics Center, George Washington University, Bethesda, MD 20817, USA; The Biostatistics Center, George Washington University, Bethesda, MD 20817, USA; Division of Nephrology, University of Washington, Seattle, WA 98195, USA; Division of Endocrinology, Columbia University Irving Medical Center, New York, NY 10032, USA; Department of Radiology and Biomedical Imaging, University of California San Francisco, San Francisco, CA 94143, USA; Bone Bioengineering Laboratory, Department of Biomedical Engineering, Columbia University, New York, NY 10027, USA; Rainbow Babies and Children’s Hospital, Case Western Reserve University, Cleveland, OH 44106, USA; Department of Epidemiology and Biostatistics, University of California San Francisco, San Francisco, CA 94158, USA

**Keywords:** epidemiology, bone QCT, aging, diabetes, bone quantification

## Abstract

The mechanism for bone fragility in type 1 diabetes (T1D) is unclear. We performed advanced analyses on HR-pQCT images from participants with T1D (*n* = 183, 59.5 ± 7.0 yr, 50.8% female, T1D duration 38.0 ± 4.8 yr) enrolled in the Epidemiology of Diabetes Interventions and Complications (EDIC) study and a demographically similar group of nondiabetic controls (*n* = 94, 60.5 ± 8.2 yr, 65.6% female). Between participants and controls, we compared trabecular morphology by individual trabecula segmentation (ITS) analysis (decomposes trabeculae into plate-like and rod-like structures), and assessed cortical topology by cortical pore skeletonization (CPS) analysis (classifies pores as slab-like or tube-like and analyzes connectivity) and by cortical laminar analysis (CLA) (quantifies distribution, number, and area of pores). By ITS, EDIC participants had worse trabecular morphology with lower plate-rod bone volume ratio (radius: 0.36 ± 0.21 vs 0.41 ± 0.25 [*p* = .031]; tibia: 0.75 ± 0.42 vs 0.88 ± 0.52 [*p* = .016]), plate tissue fraction (radius: 0.25 ± 0.10 vs 0.27 ± 0.10 [*p* = .020]; tibia: 0.40 ± 0.12 vs 0.43 ± 0.14 [*p* = .040]), and higher rod tissue fraction (radius: 0.75 ± 0.10 vs 0.73 ± 0.10 [*p* = .020]; tibia: 0.60 ± 0.12 vs 0.57 ± 0.14 [*p* = .040]) compared with controls. Epidemiology of Diabetes Interventions and Complications participants also had worse cortical pore network topology by CPS compared with controls, with increased tube-tube junctions (81.07 ± 74.84 vs 59.90 ± 45.25 [*p* = .017]) at the radius, and a higher ratio of slab-like to tube-like pores (0.490 ± 0.057 vs 0.472 ± 0.071 [*p* = .013]) at the tibia. By CLA, EDIC participants had higher pore size at midcortical (0.039 ± 0.007 vs 0.038 ± 0.007 mm^2^ [*p* = .005]) and periosteal layers (0.037 ± 0.005 vs 0.035 ± 0.006 mm^2^ [*p* < .001]), a biomechanically detrimental pattern. Among EDIC participants, higher HbA1c and advanced glycation end products, as well as kidney disease, retinopathy, and neuropathy, were associated with unfavorable trabecular morphology and cortical pore network connectivity. These findings reveal novel microarchitectural abnormalities previously associated with reduced mechanical properties of bone in participants with long-standing T1D, in association with modifiable diabetes risk factors. Trial registration: Clinicaltrials.gov NCT00360815 and NCT00360893.

## Introduction

Type 1 diabetes (T1D) is associated with an increased risk of fractures,^1^ with an up to 5-fold increase in hip fractures.^2^ As life expectancy in T1D is increasing,^3^ more individuals with T1D will live longer and be susceptible to developing fractures. Yet the mechanism for the increased fracture risk is not fully established. Individuals with T1D have reduced BMD; however, the small decrease does not explain the magnitude of the increase in fracture risk.^4^^,^^5^ Other alterations in bone quality might be contributory, with microarchitectural abnormalities playing a role.^6–8^

HR-pQCT enables detailed assessment of bone microarchitecture and geometry. This advanced imaging technique quantifies volumetric BMD along with the structural characteristics of trabecular and cortical bone compartments. When integrated with finite element analysis, these measurements yield a representation of bone strength. Because HR-pQCT-derived parameters are strong predictors of fracture risk, evaluating bone microarchitecture offers clinically valuable information that exceeds standard BMD assessments.^9^

We recently reported HR-pQCT results of bone microarchitecture and geometry in a well-phenotyped group of adults with T1D from the Epidemiology of Diabetes Interventions and Complications (EDIC) study, the observational follow-up of the Diabetes Control and Complications Trial (DCCT).^10^ By performing the standard analysis of the HR-pQCT images, we found that EDIC participants (*n* = 183) had lower BMD, larger total and trabecular area, and poorer trabecular microarchitecture as compared with controls without T1D (*n* = 94). Among participants in EDIC, lower BMD and compromised microarchitecture were associated with suboptimal glucose control, increased accumulations of advanced glycation end products (AGEs), and the presence of microvascular damage.^10^

Individual trabecula segmentation (ITS) analysis, which decomposes the trabecular network, can provide further details about plate-like (advantageous) and rod-like (disadvantageous) trabecular structures.^11^ Similarly, cortical topology can be classified using the analogous cortical pore skeletonization (CPS) analysis, which classifies individual pores as wide, slab-like pores or narrow, tube-like pores and analyzes their connectivity.^12^ These features are further explored with cortical laminar analysis (CLA), which quantifies the number and area of every cortical pore and its spatial distribution across different layers of cortical bone from endosteal to periosteal.^13^ There has been no study to date using these advanced tools in the analysis of HR-pQCT images of T1D adults. We hypothesized that deficits in HR-pQCT parameters assessed by ITS, CPS, and CLA in the DCCT/EDIC cohort would be associated with T1D and its accompanying risk factors, including poor glycemic management, accumulation of AGEs, and microvascular damage.

## Materials and methods

The DCCT/EDIC study has been previously described.^14^^,^^15^ Briefly, between 1983 and 1989, the DCCT randomly assigned 1441 participants with T1D (age range, 13-39 yr) without a history of CVD, hypertension, hyperlipidemia, or neuropathy requiring medical intervention to intensive therapy, aimed at achieving glycemia as close to the nondiabetic range as safely possible, or to conventional diabetes therapy at the time. The goal was to evaluate the effects of glycemia on the development and progression of diabetes-related complications.^14^ Two parallel cohorts were recruited: a primary prevention cohort (with 1-5 yr diabetes duration, no retinopathy, and urine albumin excretion rate [AER] <40 mg/24 h) and a secondary intervention cohort (1-15 yr duration, mild-to-moderate non-proliferative diabetic retinopathy, and AER ≤200 mg/24 h). After a mean (range) of 6.5^3–9^ yr of follow-up, intensive therapy, with a mean HbA1c of ~7%, markedly reduced the development and progression of microvascular complications compared with conventional therapy with a mean HbA1c of ~9%. At the end of the DCCT, all participants were encouraged to adopt intensive therapy and transitioned to their local health care practitioners for ongoing diabetes care. In 1994, 96% of the surviving DCCT cohort enrolled in the EDIC observational study.^15^

During EDIC years 25-26 (2018-2019), after an average follow-up of 32 yr, all surviving participants at the 27 EDIC clinics were invited to participate in the EDIC Skeletal Health ancillary study. HR-pQCT scanners were available at 6 EDIC clinical sites. HR-pQCT scans of the distal radius and tibia were performed using a second-generation scanner (XtremeCT II; Scanco Medical AG) in 183 (73%) of the 247 active EDIC participants recruited across these 6 clinics.^10^ The characteristics of this subgroup were similar to those of the overall surviving cohort at the time of acquisition. All participants gave written informed consent before enrollment, and the study protocol was reviewed and approved by the institutional review boards of all involved sites.

### Control subjects without diabetes

The ancillary study sought to enroll 100 nondiabetic controls (without either type 1 or type 2 diabetes [T2D]) from the same 6 EDIC sites.^10^ Spouses of EDIC participants, defined to include partners by legally recognized marriages, civil unions, or domestic partnerships, regardless of sex, were prioritized for recruitment. No age restrictions were applied to spousal participants. When a spouse was unavailable, recruitment efforts expanded to include other family members or friends of EDIC participants. Although sex matching was not required, each non-spousal control was matched to an EDIC participant within a 5-yr age range. In total, 103 control participants were enrolled. Of these, 94 completed second-generation HR-pQCT imaging and completed a clinic visit that involved a complete medical history and recording of current medications as well as a detailed physical examination, which included height and weight. To assess HbA1c, serum creatinine, and 25OHD levels, fasting blood samples were also collected. The range of current HbA1c among control participants was 4.7%-6.2%, below the threshold of 6.5% for diabetes.

### High-resolution peripheral quantitative computed tomography

**Figure 1:**
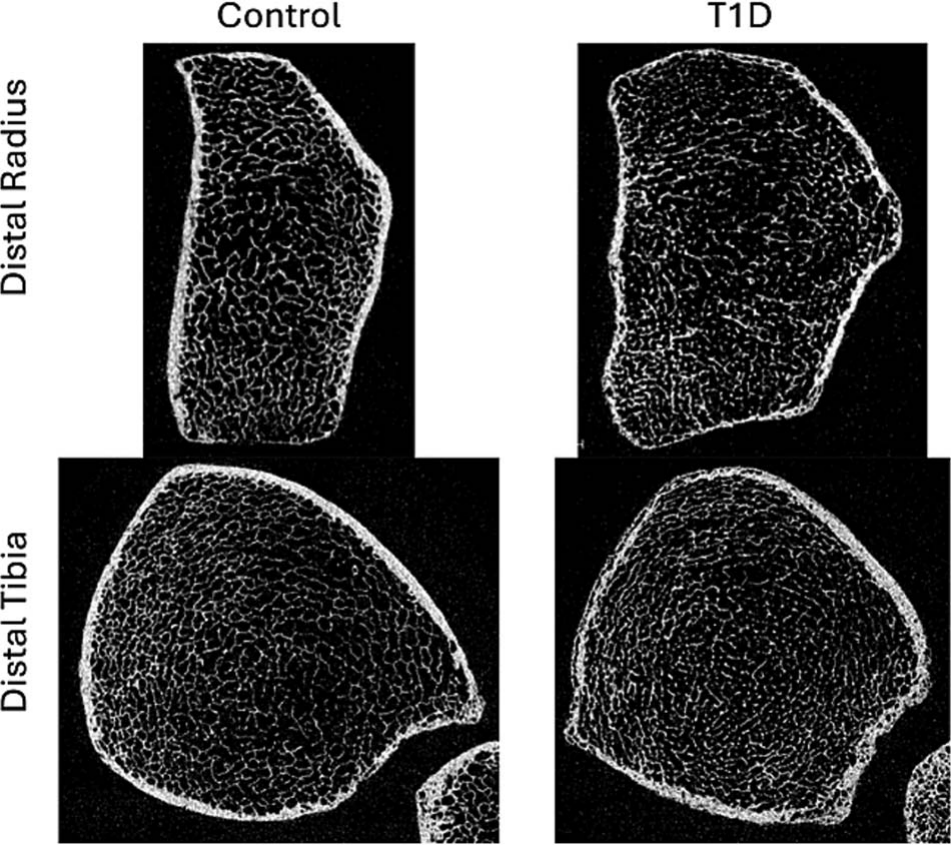
Representative cross-sectional HR-pQCT image for control subject (left) and participant with type 1 diabetes (right) at the distal radius (top) and distal tibia (bottom).

Between March 2018 and September 2019, HR-pQCT scans were obtained for each EDIC participant and control subject during a single imaging visit.^10^ As described in our earlier work, the scan region was positioned at the distal metaphysis of the radius and tibia, corresponding to offsets of 4.0% (radius) and 7.3% (tibia) of total limb length measured proximally from the distal articular surfaces.^10^ Representative cross-sectional HR-pQCT images at the distal radius and distal tibia from a control subject and participant with T1D are shown in Figure 1.

### Individual trabecular segmentation

Individual trabecular segmentation is a quantitative morphological technique designed to deconstruct the cancellous bone network into constituent plate- and rod-like elements. The methodology has been previously detailed.^11^ In summary, digital topological analysis is employed to generate a skeletonized representation of the trabecular surface, wherein each voxel is classified as part of a surface (plate) or a curve (rod) while maintaining the topological integrity and morphological characteristics of the trabecular microarchitecture.^16^ These skeletonized elements are subsequently mapped back onto the original volumetric image through an iterative reconstruction algorithm, enabling voxel-level classification into individual plates or rods. Following segmentation, a comprehensive set of ITS-derived structural parameters is computed based on the geometry and spatial orientation of each trabecula. These include plate and rod bone volume fractions (pBV/TV and rBV/TV; total volume of plates or rods/bulk volume), axial bone volume fraction (aBV/TV; bone volume aligned along the longitudinal axis), and tissue fractions (pBV/BV and rBV/BV; total volume of plates or rods/total bone volume). Additional metrics include the plate-rod tissue fraction ratio (P-R ratio), plate and rod number density (pTb.N and rTb.N, 1/mm; cubic root of total number of plates or rods/bulk volume), average plate thickness and rod diameter (pTb.Th and rTb.Th, mm), and plate-plate junction density (P-P Junc.D, 1/mm^3^; number of junctions/bulk volume). The ITS methodology has recently been validated by our group for application to second-generation HR-pQCT data through comparison with microCT, demonstrating strong concordance between modalities, with correlation coefficients (R^2^) ranging from 0.55 to 0.94. The results indicate strong correlations.^17^

### Cortical segmentation and analysis

#### Cortical segmentation

Segmentation of the cortical bone compartment was conducted using image processing language (Scanco Medical AG), employing a semiautomated 3-step algorithm, as previously described in detail.^18^ Briefly, the process began with automated contouring to delineate the periosteal and endosteal surface of the cortex; these auto-contours were reviewed and manually adjusted when necessary. In the second step, intracortical pores were identified within the cortical region. Lastly, the cortical and pore masks were integrated to generate a final, refined mask of the cortical compartment.

#### Cortical pore skeletonization

Skeletonization of cortical pores and laminar analysis were conducted using MATLAB software (MathWorks, Inc.). Cortical pore skeletonization, an analysis technique analogous to ITS but applied to the cortical pore network, has been described previously.^12^ Operating on the intracortical pore space, CPS skeletonizes pores and classifies each voxel of the skeletonized pore network as belonging to a surface or a curve. The voxel classifications are subsequently mapped onto the original pore structure, enabling the identification of each voxel as part of an individual slab-like pore (those associated with surface skeletons) or tube-like pore (those associated with curve skeletons). Tube-like pores represent osteonal Haversian canals, while slab-like pores are large interconnected canals resulting from osteonal merging and associated with increased local stress concentrations.^19^

The CPS method is adapted from ITS, applying identical skeletonization and classification steps to the inverse of the segmented HR-pQCT image, so that cortical porosity becomes the focus of analysis. This approach enables a direct comparison between trabecular and cortical pore architecture metrics. While ITS processing was conducted within the MedTool platform, CPS analysis was implemented in MATLAB to enable custom handling of the inverse images and specialized calculations for cortical pore connectivity and spatial distribution, which are not available in the base MedTool package.

Based on the pore skeletonization and classification, the total number of slab-like pores, tube-like pores, and pore network junction elements (number of tube-tube junctions and number of slab-tube junctions) was calculated. The volume of each slab and tube was calculated from the propagated image, and the mean slab volume (mm^3^) and the mean tube volume (mm^3^) were quantified. To investigate the overall structure of the cortical pore network, the following 2 ratios were calculated: total number of slabs/total number of tubes and total slab volume/total tube volume.

### Cortical laminar analysis

The segmented cortical compartment was subdivided into 3 equal-width concentric layers corresponding to the endosteal, midcortical, and periosteal laminar layers as described in detail previously.^13^^,^^20^ Then the skeletonized pore network^12^ was used to assign each pore segment to a layer based on the location of its centroid. To evaluate cortical porosity across layers, 3 quantitative metrics were derived: total pore area (expressed as mm^2^/mm^2^), total pore number, and average pore size (mm^2^). Within each cross-sectional slice of the analyzed volume, total pore area was determined by summing the areas of all pores assigned to a given layer. Total pore number was also recorded for each layer. To account for differences in layer size, specifically, the greater surface area of the periosteal layer compared with the endosteal layer, both total pore area and total pore number were normalized by the area of the respective layer. The average pore size was computed as the average size of pores within each layer. The RMSCV for laminar porosity measures ranged from 3.04% to 3.94% in the midcortical region, 4.37% to 6.09% in the periosteal, and 5.77% to 9.35% in the endosteal region.^13^

### DCCT/EDIC evaluations

Risk factors were evaluated annually during the EDIC study using standardized protocols and included self-reported data on menopausal status, medication use, smoking habits, alcohol intake, and history of clinically diagnosed fractures, along with measurements of height and weight. HbA1c was measured centrally via high-performance liquid chromatography, conducted quarterly during DCCT and annually thereafter throughout EDIC follow-up.^21^ A cumulative HbA1c value was calculated as the time-weighted average of all follow-up measures from the DCCT baseline to the time of HR-pQCT assessment. Skin intrinsic fluorescence, an indirect measure of tissue accumulation of AGEs, was measured once during EDIC years 16-17 (2009-2010), 8 yr before the HR-pQCT scans, using the SCOUT DS skin fluorescence spectrometer on the ventral side of the left forearm near the elbow.

Standardized 7-field fundus photography was performed biannually during the DCCT and annually in one quarter of the EDIC cohort, with all images graded at a central reading center.^22^ Proliferative diabetic retinopathy (PDR) was defined based on the presence of neovascularization identified through fundus photograph grading, self-reported diagnosis, or documentation of scatter photocoagulation at any point during DCCT/EDIC. The estimated glomerular filtration rate (eGFR) was derived from serum creatinine using the Chronic Kidney Disease Epidemiology Collaboration (CKD-EPI) equation. Albumin excretion rate was assessed annually during the DCCT and every other year during EDIC. From the start of DCCT through EDIC year 18, AER was measured using fluoroimmunoassay on 4-h urine collections; thereafter, it was estimated from spot urine samples based on the albumin-to-creatinine ratio.^23^ Macroalbuminuria was defined as an AER equal to or exceeding 300 mg/d. The presence/absence of each eye and kidney complication was defined as any report between DCCT baseline and the time of the bone evaluation.

Diabetic peripheral neuropathy (DPN) was evaluated once in each participant during EDIC years 13-14 (2006-2007) and was defined by the presence of confirmed clinical neuropathy, necessitating at least 2 abnormal findings, encompassing symptoms, sensory deficits, or altered reflexes, consistent with DPN as determined by a neurologist, in addition to abnormal nerve conduction in a minimum of 2 anatomically distinct nerves.^24^ Complementarily, the Michigan Neuropathy Screening Instrument (MNSI), which includes a standardized questionnaire, foot inspection, and assessment of ankle reflexes and distal vibration sensation, was administered annually throughout EDIC. A clinical examination score ≥ 2.5 on the MNSI was considered indicative of peripheral neuropathy.^25^

Serum concentrations of 25(OH)D2 and 25(OH)D3 were quantified using liquid chromatography tandem-mass spectrometry at the EDIC central biochemistry laboratory during the HR-pQCT study visit. Interassay coefficients of variability were 7.5% for 25(OH)D2 and 5.4% for 25(OH)D3, based on a mean concentration of 2 ng/mL. Total 25(OH) D was derived by summing the concentrations of 25(OH)D2 and 25(OH)D3.

### Statistical analyses

Adjusted differences between EDIC participants and control subjects, expressed as both absolute and percentage differences, were evaluated using generalized estimating equation models. These models employed an identity link function and a compound symmetry covariance structure to account for the correlation between EDIC participants and their matched control subjects. Covariate adjustment was performed for established confounders known to influence the association between diabetes and skeletal health, including age, sex, menopausal status, weight, height, 25(OH)D level, use of osteoporosis pharmacotherapy, and oral glucocorticoid exposure.

Within the EDIC cohort, separate linear regression models were utilized to examine associations between diabetes-related risk factors (including mean HbA1c) and ITS, CPS, and CLA parameters. These models were adjusted for age, sex, menopause status, weight, and height. More specifically, we analyzed (1) the effect of mean HbA1c, (2) the independent effect of the mean HbA1c and skin AGEs by including both in multivariable models, and (3) the effect of each diabetes-related complication (eg, PDR) in separate models adjusted for mean HbA1c and skin AGEs. eGFR is presented as a decrease per 20 mL/min/1.73 m2, approximately 1 SD of eGFR among EDIC participants.

All analyses were conducted using SAS statistical software (SAS Institute). Statistical significance was defined as a 2-sided *p*-value <.05. Given the observational nature of our study, no adjustment for multiple testing was applied; therefore, the findings should be interpreted with appropriate caution.

## Results

### Participant characteristics

At the time of the bone analysis by HR-pQCT, the 183 EDIC participants (50.8% female) had a mean age (± SD) of 59.5 ± 7.0 yr, T1D duration was 38.0 ± 4.8 yr, and current HbA1c was 7.8 ± 1.1%. Among the 94 control participants (65.6% female), the mean age was 60.5 ± 8.2 yr, and current HbA1c was 5.5 ± 0.3%. Self-reported history of any fracture was noted in 41.0% of EDIC participants and 31.2% of control subjects (Table 1).

**Table 1:** Characteristics of EDIC participants with type 1 diabetes and controls without diabetes with bone analysis by HR-pQCT.

	**EDIC participants (*n* = 183)**		**Control subjects (*n* = 94)**	
	*N*	Mean ± SD or N (%)	*N*	Mean ± SD or *N* (%)
**Demographics**				
** Attained age (yr)**	183	59.5 ± 7.0	94	60.4 ± 8.2
** Sex (female)**	183	93 (50.8)	94	61 (64.9)
** Postmenopausal (females only)**	93	77 (82.8)	61	36 (59.0)
** Mean menopausal age (yr)**	63	49.3 ± 5.0	29	50.7 ± 5.2
** Menopause duration** **^a^** **(yr)**	63	13.1 ± 6.6	29	11.8 ± 6.5
**Physical**				
** Height (cm)**	183	170.8 ± 9.2	93	167.0 ± 9.8
** Weight (kg)**	183	83.4 ± 18.3	93	78.5 ± 16.8
** Tibia: limb length (mm)**	180	376.1 ± 39.1	92	367.2 ± 39.3
** Radius: limb length (mm)**	163	270.5 ± 21.5	85	267.2 ± 24.3
**Self-reported history of medical conditions**				
** Fracture (ever)**	183	75 (41.0)	93	29 (31.2)
** Current smoker**	183	12 (6.6)	93	4 (4.3)
** Heavy alcohol use** **^b^**	183	11 (6.0)	93	4 (4.3)
**Diabetes-related risk factors**				
** Diabetes onset age (yr)**	183	21.6 ± 8.2		NA
** Diabetes duration (yr)**	183	38.0 ± 4.8		NA
** Mean HbA1c over DCCT/EDIC (%)**	183	8.0 ± 0.9		NA
** Current HbA1c (%)**	183	7.8 ± 1.1	92	5.5 ± 0.3
** Skin AGEs** **^c^**	179	22.3 ± 4.4		NA
**Bone biomarker**				
** 25(OH)D (ng/mL)**	183	36.2 ± 14.3	94	35.4 ± 13.5
**Current bone-active medications**				
** Osteoporosis treatment**	182	4 (2.2)	93	4 (4.3)
** Thiazide diuretic**	182	25 (13.7)	93	9 (9.7)
** Oral glucocorticoid**	182	14 (7.7)	93	0 (0.0)
** Thyroid**	180	59 (32.8)	93	12 (12.9)
** HRT (females only)**	93	6 (6.5)	61	5 (8.2)
** HRT (postmenopausal females only)**	77	5 (6.5)	36	3 (8.3)
**Microvascular complications**				
** Retinopathy**				
** Any PDR** **^d^**	183	57 (31.1)		NA
** Kidney disease**				
** eGFR (mL/min/1.73 m** ^**2**^ **)**	183	82.8 ± 20.4	92	83.8 ± 13.4
** eGFR<60 mL/min/1.73 m** ^**2**^	183	18 (9.8)	92	5 (5.4)
** Macroalbuminuria (any AER ≥300 mg/d)**	183	25 (13.7)		NA
**Peripheral neuropathy**				
** DPN** **^e^**	171	52 (30.4)		NA
** MNSI**^f^** clinical score ≥ 2.5** **^e^**	183	63 (34.4)		NA

Abbreviations: AER, albumin excretion rate; AGE, advanced glycation end product; DCCT, Diabetes Control and Complications Trial; DPN, diabetic peripheral neuropathy; EDIC, Epidemiology of Diabetes Interventions and Complications; eGFR, estimated glomerular filtration rate; HRT, hormone replacement therapy; MNSI, Michigan Neuropathy Screening Instrument; PDR, proliferative diabetic retinopathy.

HR-pQCT measured in EDIC years 25–26 (2018–2019). NA, not applicable.

a Based on the nonmissing self-reported age at menopause.

b Average consumption of any alcohol ≥27 g/d.

c AGEs measured through skin-intrinsic fluorescence in EDIC years 16–17 (2009-2010).

d PDR measured in EDIC years 18-23 (2011-2016).

e DPN measured in EDIC years 13-14 (2006-2007). ^f^MNSI measured annually during EDIC.

### Comparisons between EDIC participants and control subjects

**Figure 2:**
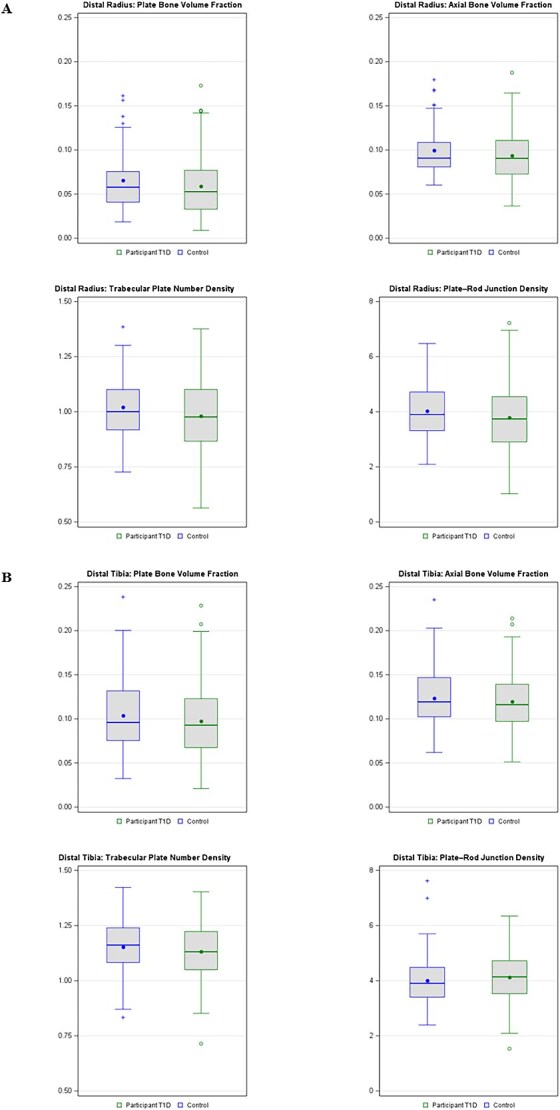
Distribution of bone microarchitecture outcomes for control subjects (green, right) and participants with type 1 Diabetes (blue, left) at the distal radius (A) and distal tibia (B).

The distributions of select bone microarchitecture outcomes for control subjects and EDIC participants at the distal radius and distal tibia are presented in Figure 2.

#### ITS parameters

Compared with control subjects, EDIC participants had significantly lower plate-rod bone volume ratio (adjusted difference radius − 0.060 [*p* = .031]; tibia −0.149 [*p* = .016]) and plate tissue fraction (radius − 0.029 [*p* = .020]; tibia −0.0351 [*p* = .040]) as well as higher rod tissue fraction (radius 0.029 [*p* = .020]; tibia 0.035 [*p* = .040]) at both the distal radius and the distal tibia (Table 2). In addition, EDIC participants also had significantly lower bone volume fraction, plate bone volume fraction, axial bone volume fraction, trabecular plate number density, mean trabecular rod thickness, plate-plate junction density, and plate-rod junction density at the distal radius, compared with control subjects.

**Table 2:** ITS parameters among EDIC participants with T1D and controls without diabetes.

	**Distal radius**			**Distal tibia**		
	**T1D (*n* = 183)**	**Controls (*n* = 94)**		**T1D (*n* = 183)**	**Controls (*n* = 94)**	
	Mean ± SD	Mean ± SD	Adjusted difference^a^ (95% CI)	Mean ± SD	Mean ± SD	Adjusted difference^a^ (95% CI)
**ITS parameters**						
** Bone volume fraction**	0.22 ± 0.05	0.23 ± 0.04	**−0.013 (−0.022, −0.004)**	0.24 ± 0.04	0.24 ± 0.03	0.00001 (−.00790, 0.00793)
** Plate bone volume fraction**	0.06 ± 0.03	0.07 ± 0.03	**−0.010 (−0.017, −0.002)**	0.10 ± 0.04	0.10 ± 0.04	−0.008 (−0.018, 0.002)
** Rod bone volume fraction**	0.17 ± 0.03	0.17 ± 0.03	−0.004 (−0.010, 0.003)	0.14 ± 0.03	0.13 ± 0.03	0.0080 (−0.0003, 0.0163)
** Plate-rod bone volume ratio**	0.36 ± 0.21	0.41 ± 0.25	**−0.060 (−0.114, −0.005)**	0.75 ± 0.42	0.88 ± 0.52	**−0.149 (−0.269, −0.028)**
** Axial bone volume fraction**	0.09 ± 0.03	0.10 ± 0.03	**−0.008 (−0.015, −0.002)**	0.12 ± 0.03	0.12 ± 0.03	−0.004 (−0.012, 0.003)
** Plate tissue fraction**	0.25 ± 0.10	0.27 ± 0.10	**−0.029 (−0.054, −0.005)**	0.40 ± 0.12	0.43 ± 0.14	**−0.035 (−0.069, −0.002)**
** Rod tissue fraction**	0.75 ± 0.10	0.73 ± 0.10	**0.029 (0.005, 0.054)**	0.60 ± 0.12	0.57 ± 0.14	**0.035 (0.002, 0.069)**
** Trabecular plate number density (1/mm)**	0.98 ± 0.17	1.02 ± 0.14	**−0.057 (−0.094, −0.021)**	1.13 ± 0.13	1.15 ± 0.12	−0.022 (−0.053, 0.009)
** Trabecular rod number density (1/mm)**	1.56 ± 0.10	1.57 ± 0.09	−0.004 (−0.028, 0.021)	1.46 ± 0.12	1.44 ± 0.14	0.033 (−0.001, 0.067)
** Mean trabecular plate thickness (mm)**	0.20 ± 0.01	0.21 ± 0.02	−0.0003 (−0.0020, 0.0015)	0.21 ± 0.01	0.21 ± 0.01	−0.001 (−0.003, 0.001)
** Mean trabecular rod thickness (mm)**	0.23 ± 0.01	0.23 ± 0.01	**−0.0022 (−0.0043, −0.0001)**	0.23 ± 0.01	0.23 ± 0.01	−0.0001 (−0.0022, 0.0020)
** Mean trabecular plate surface area (mm** ^**2**^ **)**	0.27 ± 0.01	0.28 ± 0.02	−0.003 (−0.006, 0.001)	0.30 ± 0.02	0.30 ± 0.03	−0.004 (−0.010, 0.002)
** Mean trabecular rod length (mm)**	1.00 ± 0.01	1.00 ± 0.01	−0.001 (−0.004, 0.002)	1.01 ± 0.02	1.01 ± 0.02	−0.002 (−0.006, 0.002)
** Plate-plate junction density (1/mm** ^**3**^ **)**	2.45 ± 0.95	2.64 ± 0.81	**−0.280 (−0.478, −0.082)**	3.04 ± 0.71	3.03 ± 0.64	−0.007 (−0.169, 0.155)
** Plate-rod junction density (1/mm** ^**3**^ **)**	3.79 ± 1.24	4.03 ± 0.95	**−0.351 (−0.604, −0.098)**	4.13 ± 0.91	4.01 ± 0.90	0.099 (−0.123, 0.321)
** Rod-rod junction density (1/mm** ^**3**^ **)**	3.35 ± 0.93	3.34 ± 0.85	0.054 (−0.170, 0.278)	2.53 ± 0.99	2.33 ± 1.09	0.230 (−0.038, 0.498)

Abbreviations: EDIC, Epidemiology of Diabetes Interventions and Complications; ITS, individual trabecula segmentation; T1D, type 1 diabetes.

a Differences in least squares means between EDIC participants and control subjects without diabetes obtained from separate generalized estimating equation models adjusted for age, sex, menopause status, weight, height, 25(OH)D ng/mL, osteoporosis medication, and oral glucocorticoids. Boldface indicates significance at *p* ≤ .05.

#### Cortical pore skeletonization and CLA parameters

Compared with control subjects, EDIC participants had a significantly higher number of tube-tube junctions (13.61 [*p* = .017]) at the distal radius (Table 3). Epidemiology of Diabetes Interventions and Complications participants also had significantly higher average pore size at the midcortical layer (adjusted difference 0.002 mm^2^ [*p* = .005]), periosteal layer (0.002 mm^2^ [*p* < .001]), and entire cortical compartment (0.0015 mm^2^ [*p* = .017]) as well as a higher total number of slabs to total number of tubes ratio (0.017 [*p* = .013]) at the distal tibia, compared with control subjects (Table 3).

To evaluate whether the segmentation method influenced results, we repeated the ITS and CLA analyses using the manufacturer’s standard segmentation approach. The results were consistent with those obtained using Laplace–Hamming segmentation, with the same patterns and statistical significance observed across groups (Supplemental Table S1 and S2).

**Table 3:** CPS and CLA parameters among EDIC participants with T1D and controls without diabetes.

	**Distal radius**			**Distal tibia**		
	**T1D** **(*n* = 183)**	**Controls** **(*n* = 94)**		**T1D** **(*n* = 183)**	**Controls (*n* = 94)**	
	Mean ± SD	Mean ± SD	Adjusted difference^a^ (95% CI)	Mean ± SD	Mean ± SD	Adjusted difference^a^ (95% CI)
**CLA parameters**						
** Endosteal layer**						
** Total pore area (mm** ^**2**^ **)**	0.51 ± 0.39	0.41 ± 0.26	0.053 (−0.012, 0.119)	2.75 ± 1.58	2.57 ± 1.39	−0.258 (−0.569, 0.052)
** Total pore number**	14.39 ± 12.35	11.43 ± 7.36	1.630 (−0.253, 3.514)	71.17 ± 40.57	65.35 ± 35.21	−5.981 (−13.554, 1.592)
** Average pore size (mm** ^**2**^ **)**	0.04 ± 0.01	0.04 ± 0.01	−0.00004 (−0.00122, 0.00114)	0.039 ± 0.004	0.04 ± 0.01	−0.0004 (−0.0017, 0.0009)
** Midcortical layer**						
** Total pore area (mm** ^**2**^ **)**	1.12 ± 0.58	0.93 ± 0.55	0.105 (−0.007, 0.217)	5.14 ± 1.88	4.68 ± 2.13	−0.054 (−0.492, 0.385)
** Total pore number**	32.40 ± 17.46	27.02 ± 15.03	2.817 (−0.043, 5.676)	133.07 ± 48.08	123.18 ± 47.06	−5.053 (−15.381, 5.275)
** Average pore size (mm** ^**2**^ **)**	0.04 ± 0.01	0.03 ± 0.01	0.001 (−0.001, 0.003)	0.039 ± 0.007	0.038 ± 0.007	**0.002 (0.001, 0.004)**
** Periosteal layer**						
** Total pore area (mm** ^**2**^ **)**	0.33 ± 0.28	0.25 ± 0.20	0.037 (−0.015, 0.089)	2.80 ± 1.75	2.39 ± 1.55	−0.009 (−0.387, 0.368)
** Total pore number**	9.79 ± 8.62	7.44 ± 6.04	1.052 (−0.476, 2.579)	75.47 ± 46.06	67.62 ± 41.13	−3.733 (−13.267, 5.801)
** Average pore size (mm** ^**2**^ **)**	0.03 ± 0.01	0.03 ± 0.01	0.001 (−0.001, 0.002)	0.04 ± 0.01	0.04 ± 0.01	**0.002 (0.001, 0.004)**
** Entire compartment**						
** Total pore area (mm** ^**2**^ **)**	1.96 ± 1.12	1.58 ± 0.96	0.194 (−0.007, 0.395)	10.69 ± 4.64	9.64 ± 4.59	−0.341 (−1.345, 0.663)
** Total pore number**	56.58 ± 35.29	45.89 ± 27.51	5.559 (−0.131, 11.249)	279.7 ± 128.2	256.2 ± 116.1	−14.866 (−40.332, 10.600)
** Average pore size (mm** ^**2**^ **)**	0.04 ± 0.01	0.034 ± 0.004	0.001 (−0.001, 0.002)	0.04 ± 0.01	0.04 ± 0.01	**0.0015 (0.0003, 0.0027)**
**CPS Parameters**						
** Number of slab-like pores**	439 ± 319	350 ± 226	47 (−3, 96)	2597 ± 1260	2337 ± 1142	−70 (−329, 189)
** Number of tube-like pores**	1112 ± 740	903 ± 549	105 (−1, 211)	5328 ± 2573	4908 ± 2244	−281 (−771, 208)
** Number of tube-tube junctions**	81 ± 75	60 ± 45	**14 (2, 25)**	586 ± 352	524 ± 325	−25 (−99, 50)
** Number of slab-tube junctions**	767 ± 579	606 ± 410	85 (−6, 177)	5031 ± 2575	4508 ± 2345	−131 (−664, 401)
** Total # slabs/total # tubes**	0.39 ± 0.06	0.38 ± 0.06	0.006 (−0.010, 0.022)	0.49 ± 0.06	0.47 ± 0.07	**0.017 (0.004, 0.031)**
** Total slab volume/total tube volume**	0.23 ± 0.11	0.20 ± 0.08	0.013 (−0.011, 0.037)	0.34 ± 0.10	0.32 ± 0.11	0.021 (−0.007, 0.049)
** Mean volume of slab-like pores (mm** ^**3**^ **)**	0.007 ± 0.003	0.006 ± 0.002	0.0006 (−.0001, 0.0014)	0.009 ± 0.003	0.009 ± 0.004	0.0004 (−0.0005, 0.0013)
** Mean volume of tube-like pores (mm** ^**3**^ **)**	0.012 ± 0.002	0.012 ± 0.002	0.0004 (−0.0001, 0.0010)	0.014 ± 0.002	0.013 ± 0.002	0.0004 (−0.0001, 0.0008)

Abbreviations: CLA, Cortical Laminar Analysis; CPS, Cortical Pore Skeletonization; EDIC, Epidemiology of Diabetes Interventions and Complications; ITS, individual trabecula segmentation; T1D, type 1 diabetes.

a Differences in least squares means between EDIC participants and control subjects without diabetes obtained from separate generalized estimating equation models adjusted for age, sex, menopause status, weight, height, 25(OH)D ng/mL, osteoporosis medication, and oral glucocorticoids. Boldface indicates significance at *p* ≤ .05.

### Diabetes-related factors for bone deficits

Among EDIC participants, mean HbA1c was associated with several ITS parameters at the distal radius and tibia, but not CPS or CLA parameters (Table 4). More specifically, mean HbA1c was associated with lower axial bone volume fraction, plate-plate junction density, and plate-rod junction density at the distal radius (Table 5**, Model 1**) but not with plate tissue fraction or rod tissue fraction. Upon adjustment for skin AGEs, these associations were reduced and no longer statistically significant (**Model 2**), indicating that skin AGEs may mediate the relationships between mean HbA1c and microstructural parameters at the radius. Skin AGEs were associated with lower plate-plate junction density and plate-rod junction density, independent of mean HbA1c (**Model 2**). At the distal tibia, mean HbA1c was associated with lower axial bone volume fraction, plate tissue fraction, rod tissue fraction, and plate-plate junction density (**Model 1**). After adjusting for skin AGEs, the significant associations with mean HbA1c were reduced and no longer reached statistical significance (**Model 2**); however, skin AGEs were associated with each of these parameters, independent of mean HbA1c (**Model 2**).

**Table 4:** Associations of mean HbA1c with ITS, CPS, and CLA parameters among EDIC participants.

	**Distal radius**	**Distal tibia**
	**β ± SE**	**β ± SE**
**ITS parameters**		
** Bone volume fraction**	**−0.009 ± 0.004**	**−0.007 ± 0.003**
** Plate bone volume fraction**	−0.005 ± 0.003	**−0.008 ± 0.003**
** Rod bone volume fraction**	−0.004 ± 0.002	0.001 ± 0.003
** Plate-rod bone volume ratio**	−0.016 ± 0.018	−0.065 ± 0.035
** Trabecular plate number density (1/mm)**	**−0.029 ± 0.013**	**−0.028 ± 0.011**
** Trabecular rod number density (1/mm)**	−0.003 ± 0.009	0.008 ± 0.010
** Mean trabecular plate thickness (mm)**	−0.001 ± 0.001	**−0.002 ± 0.001**
** Mean trabecular rod thickness (mm)**	**−0.002 ± 0.001**	**−0.001 ± 0.001**
** Mean trabecular plate surface area (mm** ^**2**^ **)**	−0.0004 ± 0.0012	**−0.004 ± 0.002**
** Mean trabecular rod length (mm)**	0.001 ± 0.001	0.001 ± 0.001
** Rod-rod junction density (1/mm** ^**3**^ **)**	−0.010 ± 0.081	0.107 ± 0.083
**CLA parameters**		
** Endosteal layer: total pore area (mm** ^**2**^ **)**	0.01 ± 0.03	−0.13 ± 0.10
** total pore number**	0.27 ± 0.94	−2.18 ± 2.50
** average pore size (mm** ^**2**^ **)**	−0.0003 ± 0.0004	−0.0006 ± 0.0004
** Midcortical layer: total pore area (mm** ^**2**^ **)**	0.05 ± 0.04	0.10 ± 0.14
** total pore number**	1.94 ± 1.23	0.05 ± 3.10
** average pore size (mm** ^**2**^ **)**	−0.0004 ± 0.0006	0.0006 ± 0.0005
** Periosteal layer: total pore area (mm** ^**2**^ **)**	0.02 ± 0.02	0.06 ± 0.12
** total pore number**	0.67 ± 0.63	−0.79 ± 3.05
** average pore size (mm** ^**2**^ **)**	−0.0003 ± 0.0006	0.0008 ± 0.0004
** Entire compartment: total pore area (mm** ^**2**^ **)**	0.08 ± 0.08	0.04 ± 0.31
** total pore number**	2.87 ± 2.44	−2.92 ± 7.95
** average pore size (mm** ^**2**^ **)**	−0.0003 ± 0.0004	0.0003 ± 0.0003
**CPS parameters**		
** Number of slab-like pores**	29.36 ± 22.68	−9.30 ± 80.81
** Number of tube-like pores**	61.42 ± 47.99	−77.90 ± 151.88
** Total # slabs/total # tubes**	−0.004 ± 0.005	0.003 ± 0.004
** Mean volume of slab-like pores (mm** ^**3**^ **)**	−0.0001 ± 0.0003	0.0002 ± 0.0003
** Mean volume of tube-like pores (mm** ^**3**^ **)**	−0.0002 ± 0.0002	0.0001 ± 0.0001

Abbreviations: CLA, Cortical Laminar Analysis; CPS, Cortical Pore Skeletonization; EDIC, Epidemiology of Diabetes Interventions and Complications; ITS, individual trabecula segmentation; T1D, type 1 diabetes.

Data are β-estimates ± SEs from separate linear regression models adjusted for age, sex, menopause status, weight, and height. Boldface indicates significance at *p* ≤ .05. Note that the associations of mean HbA1c with the primary 5 ITS, 4 CLA, and 3 pore skeletonization parameters are listed in Tables 5 and 6 (Model 1) and not presented in Table 4.

**Table 5:** Associations of diabetes-related factors with ITS parameters among EDIC participants.

	**Axial bone volume fraction**	**Plate tissue fraction**	**Rod tissue fraction**	**Plate-plate junction density (1/mm** ^**3**^ **)**	**Plate-rod junction density (1/mm** ^**3**^ **)**
**Distal radius**					
** Model 1:**					
** Mean HbA1c (per 1%)**	**−0.005 ± 0.002**	−0.011 ± 0.008	0.011 ± 0.008	**−0.156 ± 0.074**	**−0.197 ± 0.095**
** Model 2:**					
** Mean HbA1c (per 1%)**	−0.004 ± 0.002	−0.008 ± 0.009	0.008 ± 0.009	−0.105 ± 0.077	−0.130 ± 0.099
** Skin AGEs (per 5 units)**	−0.004 ± 0.003	−0.011 ± 0.010	0.011 ± 0.010	**−0.179 ± 0.085**	**−0.246 ± 0.108**
** Models 3-7:**					
** Any PDR**	−0.005 ± 0.005	−0.021 ± 0.018	0.021 ± 0.018	−0.065 ± 0.160	−0.010 ± 0.204
** eGFR** ^**a**^	**−0.007 ± 0.003**	−0.008 ± 0.012	0.008 ± 0.012	−0.173 ± 0.104	−0.248 ± 0.132
** Any AER ≥300 mg/d**	−0.012 ± 0.007	−0.045 ± 0.026	0.045 ± 0.026	−0.125 ± 0.227	0.016 ± 0.290
** DPN**	0.0004 ± 0.0051	0.007 ± 0.018	−0.007 ± 0.018	−0.095 ± 0.159	−0.222 ± 0.203
** MNSI ≥2.5**	0.0003 ± 0.0049	0.010 ± 0.018	−0.010 ± 0.018	0.024 ± 0.153	−0.003 ± 0.196
**Distal tibia**					
** Model 1:**					
** Mean HbA1c (per 1%)**	**−0.007 ± 0.002**	**−0.022 ± 0.010**	**0.022 ± 0.010**	**−0.144 ± 0.056**	−0.116 ± 0.072
** Model 2:**					
** Mean HbA1c (per 1%)**	−0.004 ± 0.003	−0.014 ± 0.010	0.014 ± 0.010	−0.102 ± 0.059	−0.090 ± 0.076
** Skin AGEs (per 5 units)**	**−0.009 ± 0.003**	**−0.029 ± 0.011**	**0.029 ± 0.011**	**−0.160 ± 0.064**	−0.116 ± 0.083
** Models 3-7:**					
** Any PDR**	−0.007 ± 0.005	**−0.044 ± 0.021**	**0.044 ± 0.021**	−0.056 ± 0.121	0.084 ± 0.157
** eGFR** ^**a**^	−0.003 ± 0.003	0.002 ± 0.014	−0.002 ± 0.014	**−0.162 ± 0.079**	**−0.258 ± 0.102**
** Any AER ≥300 mg/d**	−0.007 ± 0.007	−0.026 ± 0.030	0.026 ± 0.030	−0.041 ± 0.166	−0.038 ± 0.216
** DPN**	0.001 ± 0.005	0.023 ± 0.021	−0.023 ± 0.021	−0.158 ± 0.118	**−0.343 ± 0.150**
** MNSI ≥2.5**	−0.005 ± 0.005	−0.006 ± 0.020	0.006 ± 0.020	−0.139 ± 0.111	−0.163 ± 0.144

Abbreviations: AER, albumin excretion rate; AGE, advanced glycation end product; DPN, diabetic peripheral neuropathy; EDIC, Epidemiology of Diabetes Interventions and Complications; eGFR, estimated glomerular filtration rate; ITS, individual trabecula segmentation; MNSI, Michigan Neuropathy Screening Instrument; PDR, proliferative diabetic retinopathy.

Data are β-estimates ± SEs from separate linear regression models, further adjusted for age, sex, menopause status, weight, and height. Boldface indicates significance at *p* ≤ .05. Models 3-7: one row per complication (yes vs. no) adjusted for mean HbA1c and skin AGEs.

a eGFR per −30 mL/min/1.73m^2^.

Models for individual complications were adjusted for mean HbA1c and skin AGEs (Table 5**, Models 3-7**). Lower eGFR was associated with lower axial bone volume fraction at the distal radius and with lower plate-plate junction density and plate-rod junction density at the distal tibia. In addition, the presence of PDR was associated with lower plate tissue fraction, while the presence of DPN was associated with lower plate-rod junction density, both at the distal tibia.

Skin AGEs were associated with lower total pore area at the endosteal layer at the distal tibia, independent of mean HbA1c (Table 6**, Model 2**). In addition, the presence of macroalbuminuria was associated with a higher number of tube-tube junctions at the distal radius, while the presence of DPN, defined by an abnormal MNSI examination, was associated with a higher number of tube-tube junctions at the distal tibia.

**Table 6:** Associations of diabetes-related factors with CPS and CLA parameters among EDIC participants.

	**Endosteal layer**	**Midcortical layer**	**Periosteal layer**	**Entire compartment**	**Number of tube-tube junctions**	**Number of slab-tube junctions**	**Total slab volume/total tube volume**
	**Total pore area (mm** ^**2**^ **)**						
**Distal radius**							
** Model 1:**							
** Mean HbA1c (per 1%)**	0.01 ± 0.03	0.05 ± 0.04	0.02 ± 0.02	0.08 ± 0.08	8.02 ± 5.46	52.01 ± 41.55	−0.0001 ± 0.0096
** Model 2:**							
** Mean HbA1c (per 1%)**	0.02 ± 0.03	0.04 ± 0.05	0.02 ± 0.02	0.08 ± 0.09	8.52 ± 5.78	51.02 ± 43.96	−0.004 ± 0.010
** Skin AGEs (per 5 units)**	−0.01 ± 0.04	0.06 ± 0.05	0.003 ± 0.025	0.05 ± 0.10	−1.53 ± 6.41	8.19 ± 48.73	0.020 ± 0.011
** Models 3–7:**							
** Any PDR**	0.07 ± 0.07	−0.09 ± 0.10	0.04 ± 0.05	0.02 ± 0.18	17.64 ± 11.85	99.50 ± 90.44	0.011 ± 0.021
** eGFR** ^**a**^	−0.05 ± 0.04	−0.07 ± 0.06	0.001 ± 0.030	−0.12 ± 0.12	−2.11 ± 7.74	−50.02 ± 58.78	0.009 ± 0.013
** Any AER ≥300 mg/d**	0.02 ± 0.10	0.11 ± 0.14	0.08 ± 0.07	0.20 ± 0.26	**33.46 ± 16.70**	178.65 ± 127.90	0.003 ± 0.029
** DPN**	0.05 ± 0.07	0.08 ± 0.10	0.09 ± 0.05	0.22 ± 0.18	7.27 ± 12.14	72.36 ± 92.19	0.036 ± 0.021
** MNSI ≥2.5**	0.03 ± 0.06	0.13 ± 0.09	0.05 ± 0.04	0.20 ± 0.17	5.09 ± 11.32	35.50 ± 86.14	0.027 ± 0.019
**Distal tibia**							
** Model 1:**							
** Mean HbA1c (per 1%)**	−0.13 ± 0.10	0.10 ± 0.14	0.06 ± 0.12	0.04 ± 0.31	5.17 ± 22.59	11.27 ± 167.25	0.007 ± 0.009
** Model 2:**							
** Mean HbA1c (per 1%)**	−0.04 ± 0.11	0.08 ± 0.15	0.10 ± 0.13	0.15 ± 0.33	8.98 ± 23.91	49.68 ± 176.95	0.006 ± 0.009
** Skin AGEs (per 5 units)**	**−0.29 ± 0.12**	0.10 ± 0.16	−0.11 ± 0.14	−0.30 ± 0.36	−8.78 ± 25.96	−92.20 ± 192.10	0.006 ± 0.010
** Models 3-7:**							
** Any PDR**	−0.32 ± 0.22	−0.43 ± 0.30	−0.11 ± 0.27	−0.86 ± 0.69	−15.34 ± 49.93	−247.15 ± 369.14	−0.015 ± 0.019
** eGFR** ^**a**^	−0.04 ± 0.15	−0.05 ± 0.20	−0.13 ± 0.18	−0.21 ± 0.45	1.06 ± 32.37	−84.08 ± 239.47	−0.016 ± 0.012
** Any AER ≥300 mg/d**	−0.25 ± 0.30	−0.04 ± 0.41	−0.06 ± 0.37	−0.35 ± 0.93	21.81 ± 67.48	−97.19 ± 499.45	−0.026 ± 0.026
** DPN**	−0.01 ± 0.22	−0.04 ± 0.30	0.26 ± 0.27	0.20 ± 0.68	−34.68 ± 49.39	−222.15 ± 365.10	0.004 ± 0.019
** MNSI ≥2.5**	0.21 ± 0.21	0.13 ± 0.28	0.30 ± 0.25	0.64 ± 0.63	**97.44 ± 45.19**	472.99 ± 337.10	−0.020 ± 0.017

Abbreviations: AER, albumin excretion rate; AGE, advanced glycation end product; DPN, diabetic peripheral neuropathy; EDIC, Epidemiology of Diabetes Interventions and Complications; eGFR, estimated glomerular filtration rate; MNSI, Michigan Neuropathy Screening Instrument; PDR, proliferative diabetic retinopathy.

Data are β-estimates ± SEs from separate linear regression models, further adjusted for age, sex, menopause status, weight, and height. Boldface indicates significance at *p* ≤ .05. Models 3-7: one row per complication (yes vs. no) adjusted for mean HbA1c and skin AGEs.

a eGFR per −30 mL/min/1.73m^2^.

## Discussion

In the DCCT/EDIC cohort, the largest T1D population to date with HR-pQCT assessments, we found deficits in trabecular and cortical bone microstructure by advanced analyses. Trabecular morphology, spatial distribution of cortical pore size, and topology of the cortical pore network in these adults with a long duration of T1D differed from similarly aged control subjects without diabetes. Epidemiology of Diabetes Interventions and Complications participants had worse trabecular morphology, with decreased plate tissue fraction and axial alignment, in association with increased AGEs and microvascular disease. They also had an increase in cortical pore size in the midcortical and periosteal layers, a biomechanically detrimental pattern, as well as worse cortical pore network topology, with increased pore junctions and a higher ratio of slab-like pores to tube-like pores, and higher tube-tube junctions in association with microalbuminuria and neuropathy. These findings reveal new microarchitectural abnormalities in association with diabetes risk factors that until now have not been reported in individuals with long-standing T1D.

Individual trabecula segmentation is an approach to characterizing the morphology of trabecular bone from HR-pQCT images that is not available with standard HR-pQCT analysis. The technique decomposes the trabecular network into individual plates and rods,^11^ so their volume, number, thickness, orientation, and connectivity can be quantified separately. Plate-like trabeculae are more efficient at bearing compressive loads and distributing mechanical forces across a larger volume of bone, providing more stability under compressive stress.^11^^,^^26^ Rod-like trabeculae, in contrast, are more slender and contribute less to load distribution.^11^^,^^26^ The application of ITS to HR-pQCT and μCT images has demonstrated how plate and rod configurations affect whole bone mechanical properties and how trabecular microstructure changes in the context of various musculoskeletal diseases.^27–30^ Until now, ITS has not been used to study adults with T1D.

In this study, we found unfavorable alterations in plate and rod structure in EDIC participants as compared with controls. Specifically, at both the radius and the tibia, EDIC participants had reduced plate tissue fraction and plate-rod bone volume, along with increased rod-tissue fraction. Additional detrimental findings were present at just the radius, including reduced axial alignment and a decrease in the volume, density and connectivity of plate-type trabeculae. These types of inferior trabecular morphologic measurements have been previously shown to correlate with worse measures by μCT,^17^ the gold standard for microstructure, and with decreased mechanical competence.^11^ In particular, the reduced plate tissue fraction and decreased axial alignment that we see in the EDIC participants correlated strongly with prior studies of subjects with reduced bone strength.^31^ Interestingly, in T2D, studies have shown that trabecular morphology might be *better* in T2D postmenopausal women as compared with controls, although only in those with shorter diabetes duration.^27^ In T2D women with fractures, ITS measures were worse than in T2D women without fractures,^32^ suggesting that these parameters might be risk factors for fracture. Overall, the reduced trabecular plate-like qualities in EDIC might help to explain the well-established increased fracture risk in T1D patients.^1^^,^^2^

Among EDIC participants, we examined which diabetes-risk factors were associated with trabecular morphological deficits. Mean HbA1c levels were inversely associated with numerous ITS parameters. Interestingly, these relationships did not persist once skin AGEs and HbA1c levels were included in our models. Rather, we found that increased skin AGEs were associated with worse ITS parameters, independently of mean HbA1c. Taken together, these findings suggest first that the effect of poorer glycemic control on ITS parameters manifests, at least in part, through the pathway of higher AGEs, and second that skin AGE levels may have a pathogenic effect on bone beyond the cumulative effects of hyperglycemia. This association is in line with our recent finding that skin AGEs are a risk factor for trabecular microarchitectural deficits by standard HR-pQCT analysis in T1D, independent of mean HbA1c.^10^ Mechanistically, AGEs are known to have an adverse impact on the mechanosensitivity of osteocytes, leading to a negative effect on their regulation of bone remodeling under mechanical stimulation.^33^ We speculate that osteocyte impairment by AGEs could reduce the signaling needed to maintain plate-like structures. Plate-like trabeculae require more mechanical adaptation and signaling to remodel^11^^,^^26^; with increased AGEs, we hypothesize that the bone might shift toward rod-like trabeculae, which bear less load and require less mechanical adaptation and signaling as compared with plates.^11^^,^^26^ This might explain the greater number of ITS deficits that we measured at the radius: perhaps more weight-bearing at the tibia partially mitigates the AGE-mediated impairment of osteocytes there, as opposed to at the non-weight-bearing radius. Although HbA1c and skin AGEs both reflect glycation processes, they capture different aspects of metabolic exposure: HbA1c reflects glycation of hemoglobin over ~3 mo, whereas tissue AGEs represent cumulative glycation and lipoxidation. Lipid levels and lipid-lowering therapy may contribute to AGE accumulation and could partially explain the associations observed between and skin AGEs and bone outcomes, independent of HbA1c.

Diabetes microvascular disease, including lower eGFR, the presence of proliferative retinopathy, and peripheral neuropathy, was associated with various measures of worse trabecular morphology after adjustment for mean HbA1c and skin AGEs. Prior HR-pQCT data with standard analyses have suggested a link between microvascular disease and T1D bone disease.^8^ It is conceivable that decreased skeletal blood supply could compromise bone remodeling such that bone resorption is favored over bone formation,^34^ perhaps leading to fewer plates and more rods. The differential associations between trabecular parameters and HbA1c versus specific microvascular complications (eg, bone volume fraction was related to HbA1c but not with complications, while plate tissue fracture was associated with PDR but not nephropathy or neuropathy) may reflect the distinct and overlapping contributions of chronic hyperglycemia and complication-specific pathophysiology. Microvascular complications represent the cumulative effects of both glycemic and non-glycemic factors (eg, genetics, comorbidities, local vascular factors), whereas HbA1c reflects glycemia alone. In addition, specific trabecular parameters may be influenced by mechanisms independent of microvascular injury, such as alterations in bone remodeling due to inflammatory mediators, insulin deficiency, or changes in mechanical loading. Similarly, some microvascular complications (eg, proliferative retinopathy) may be more closely linked to systemic processes that secondarily affect bone microarchitecture than others.

In addition to applying ITS analysis to the HR-pQCT images, we also examined the scans for the characteristics and spatial distribution of cortical bone pores by CLA. This method calculates cortical pore number and size within the endosteal, midcortical, and periosteal regions of the cortex at the distal radius and tibia, enabling quantification of the number and area of pores in each of these specified cortical layers. Despite our previous finding that the percent of overall cortical porosity did not differ between EDIC participants and controls,^10^ here we found differences by CLA in the spatial distributions of average cortical pore size at the tibia. While in the endosteal layer, there was no difference between EDIC participants and controls, the average pore size was increased in EDIC participants in the midcortical and periosteal layers, and overall in the entire compartment.^35^ This increase in average pore size detected by CLA, without a corresponding change in overall cortical porosity in our prior standard analysis,^10^ might reflect fewer but larger pores or coalescence of smaller pores in EDIC participants.^19^ These changes in pore size and location are reminiscent of previous observations in T2D.^36^ The magnitude of increase in average tibial pore size in EDIC participants of 1500 μm^2^, or 4%, could conceivably lead to increased stress concentrations and accelerated crack propagation.^35^ The site specificity of the layers has biological relevance because it means that cortical porosity in EDIC participants was not due to endosteal changes such as trabecularization with marrow cavity expansion, but perhaps to expansion of the vascular network in the midcortical and periosteal zones. Our spatial findings also have mechanical importance because pores located closer to the periosteal surface have a more detrimental effect on bone mechanical properties than those located closer to the marrow cavity.^37^ Since cortical fractures originate at microcracks near areas of more cortical porosity^38^ and bending stresses are greatest at periosteal surfaces,^39^ the higher midcortical and periosteal pore sizes in EDIC participants may help explain the higher skeletal fragility in individuals with T1D.

Among EDIC participants, we examined which diabetes-risk factors were associated with the pore size spatial distribution. Higher skin AGEs were associated at the tibia with decreased endosteal pore size, but not midcortical or periosteal pore size; this seemingly beneficial effect of AGEs is hard to explain, although perhaps any paradoxically stabilizing effect of AGEs is lost in midcortical and periosteal layers. Interestingly, the pore size spatial distribution in EDIC is reminiscent of the pattern previously reported in T2D: higher porosity in the midcortical and periosteal layers was seen in postmenopausal T2D subjects with fragility fractures versus those without fracture^36^; these similarities might suggest overlapping etiologies of pore spatial distribution among individuals with T1D and T2D. Mechanistically, the pore size spatial distribution might be explained by cortical diabetic microangiopathy, or expansion of the vascular network in the midcortical and periosteal layers, perhaps due to neovascularization triggered by ischemia. This might be consistent with the fact that we only saw pore size increases at the tibia, as diabetic microvascular disease is typically worse in the lower (vs. upper) extremities, given that the lower extremities are farther from the heart with a greater reduction in blood flow and a greater increase in small vessel disease.

To further evaluate microstructural changes within the cortex, we also performed CPS, a topological analysis of the cortical pore network, which classifies individual pores as slab-like or tube-like and analyzes their connectivity.^12^ We found that the number of tube-tube junctions at the radius was increased, while the ratio of total slabs to total tubes was increased at the tibia, both of which represent worse network topology in EDIC participants. Increased tube-tube junctions reflect merging of osteonal canals, producing a more interconnected network of pores that will have negative effects on bone mechanics. Clusters of remodeling and merging osteons are in fact more prevalent in fracture cases.^40^^,^^41^ Slab-like pores represent clusters of merged osteons, or “giant canals” that serve as focal stress concentrations and potential sites of crack initiation.

Among EDIC participants, the higher pore tube-tube junctions at the radius were associated with higher urinary albumin excretion, after adjustment for mean HbA1c and AGEs. This is consistent with the established effect of chronic kidney disease to increase cortical porosity.^42^ We speculate that secondary hyperparathyroidism, in the setting of kidney dysfunction, might promote more tube-tube junctions, particularly at the radius, where the protective effect of weight-bearing is absent. In contrast, at the tibia, higher tube-tube junctions were associated with peripheral neuropathy. This is in line with previous reports showing that peripheral neuropathy in individuals with T1D is associated with increased cortical porosity, specifically at the tibia.^6^ We speculate that tube-tube junctions are increased at the tibia as a result of altered neurovascular regulation of bone remodeling,^43^ which might be more evident distally at the tibia, given that peripheral neuropathy is a length-dependent process.

Our study has several strengths. To our knowledge, this is the first study to investigate trabecular and cortical microstructure using ITS, CPS, and CLA, with consequent insights into mechanisms of bone fragility in adults with T1D. Our analysis was done in the largest HR-pQCT study of T1D participants to date and included comprehensive data on prolonged exposure to diabetes-associated risk factors and complications, including time-weighted mean HbA1c as a measure of cumulative glycemic exposure over time, assessment of AGE accumulation, and evaluation of microvascular complications of the eyes, kidneys, and nerves. It also included standardized HR-pQCT acquisition across 6 EDIC sites, the use of advanced new-generation scanners, and a central reading site for all skeletal measures, including the ITS, CPS, and CLA parameters. An additional strength is our use of the Laplace-Hamming binarization approach, as opposed to the standard segmentation approach (shown in Supplemental Material), for our primary analysis. The Laplace-Hamming approach provides improved accuracy in segmentation of the fine details in both the trabecular and cortical compartments.^44^ Moreover, the standard segmentation technique relies on a thresholding approach based directly on BMD to segment mineralized bone from non-mineralized tissues. Importantly, T1D is a disease state that is known to have altered bone mineralization,^45^ so use of the standard approach could have introduced bias and potentially overestimated thick structures and underestimated or omitted finer structures.

Limitations of our study include the resolution of the technologies used. HR-pQCT represents one of the highest-resolution imaging modalities currently available for in vivo assessment. However, the HR-pQCT image voxel size (60.7 μm) is on the order of the thickness of an individual trabecula, so the ITS procedure could mistakenly break up a plate-like trabecula into several, smaller rod-like trabecula during decomposition, leading to an underestimation of plate parameters and overestimation of rod parameters and a falsely more disconnected structure. In addition, bone can either be present or absent in a particular voxel, leading to partial volume effects and an erroneously increased trabecula thickness since thresholding cannot account for part of a voxel containing bone. Similarly, HR-pQCT is limited in its ability to detect pores with diameters smaller than approximately 100 μm.^13^ However, pores of larger diameter are resolved, and it is these larger ones that are the primary contributors to altered mechanical properties and risk of fragility fractures.^46^ Moreover, limitations in our technologies would have equally impacted both EDIC participants and control subjects. An additional limitation is that we only used a single cross-sectional HR-pQCT assessment, which prevented analysis of temporal associations between T1D and bone outcomes. Additionally, skin AGEs were only measured once, approximately 8 yr before the HR-pQCT scans being obtained. Additionally, skin autofluorescence may not fully reflect AGE accumulation in bone.

The study’s findings may not be fully generalizable to all individuals with T1D, especially since the cohort’s age range (44-74 yr) is not yet at the highest fracture risk, and diabetes management has improved significantly over the past 30 yr. Moreover, it is possible that our results pertain to the peripheral skeleton and might not be representative of the microarchitecture at the axial skeleton. Control subjects, who are spouses or significant others of EDIC participants, may have also adopted healthier lifestyles than the general population without diabetes. Given the exploratory nature of these analyses, adjustments for multiple comparisons were not made; therefore, the findings should be interpreted with caution. Finally, the EDIC group had fewer women, most of whom were postmenopausal, compared with the control group; however, we adjusted for these differences in all our analyses.

In conclusion, advanced analyses of HR-pQCT images revealed that long-standing T1D is associated with deficits in trabecular and cortical bone microstructure that have not been previously reported. These include reduced plate tissue fraction and axial alignment of trabecula, in association with poorer glycemic control, increased AGEs, and microvascular disease. They also include increased cortical pore size at the mechanically important midcortical and periosteal layers and altered cortical pore network topology in association with microalbuminuria and neuropathy. We acknowledge that statistically significant findings do not necessarily imply clinical significance. However, even relatively small structural differences in trabecular bone have been associated in prior studies with meaningful differences in bone strength and fracture risk, particularly when occurring alongside other microarchitectural deficits. Therefore, these results may suggest potentially meaningful patterns that might help to explain the well-established increased fracture risk in T1D patients. Efforts to maintain glycemic control, reduce AGE accumulation, and prevent microvascular disease may preserve trabecular and cortical microstructure and potentially reduce future fracture risk in aging adults with long-standing T1D.

## Supplementary Material

SH5_ITS_Cortical_Laminar_Supplementary_Material_ziaf167

## Data Availability

Data collected for the DCCT/EDIC study through June 30, 2017 are available to the public through the NIDDK Central Repository (https://repository.niddk.nih.gov/studies/edic/). Data collected in the current cycle (July 2017-June 2022) will be available within two years after the end of the funding cycle.
