# Supplementary material for: Bone microarchitecture assessed by High-resolution peripheral quantitative computed tomography in type 1 diabetes: the Diabetes Control and Complications Trial/Epidemiology of Diabetes Interventions and Complications study
Source: JBMR Plus. 2025 Oct 22;9(12):ziaf167. doi: 10.1093/jbmrpl/ziaf167 (PMC12607258; doi:10.1093/jbmrpl/ziaf167)
Supplement: SH5_ITS_Cortical_Laminar_Supplementary_Material_ziaf167 [file sh5_its_cortical_laminar_supplementary_material_ziaf167.docx]

**Online-only Supplemental Materials**

**Title**: Bone Microarchitecture Assessed by HR-pQCT in Type 1 Diabetes: The DCCT/EDIC Study

**Running Title**: ITS and Cortical Laminar Analysis in T1D

Mishaela Rubin, MD^*^, Galateia Kazakia, PhD^*^, Barbara H. Braffett, PhD, Naina Sinha Gregory, MD, Ming-Hui Lin, MPH, Victoria Trapani, MPH, Ian H. de Boer, MD, Sanchita Agarwal, MS, Andrew Burghardt, X. Edward Guo, PhD, Rose Gubitosi-Klug, MD, PhD, Ann Schwartz, PhD, for the DCCT/EDIC Research Group

*Co-first authors

**Table S1. ITS Parameters among EDIC Participants with T1D and Controls without Diabetes**

|  | **Distal Radius** | | | **Distal Tibia** | | |
| --- | --- | --- | --- | --- | --- | --- |
|  | **T1D (n=183)** | **Controls (n=94)** |  | **T1D (n=183)** | **Controls (n=94)** |  |
|  | Mean ± SD | Mean ± SD | Adjusted difference*  (95% CI) | Mean ± SD | Mean ± SD | Adjusted difference*  (95% CI) |
| **ITS Parameters** |  |  |  |  |  |  |
| Bone volume fraction | 0.20 ± 0.06 | 0.21 ± 0.05 | **-0.018 (-0.030, -0.005)** | 0.23 ± 0.05 | 0.23 ± 0.05 | -0.004 (-0.016, 0.007) |
| Plate bone volume fraction | 0.08 ± 0.04 | 0.08 ± 0.04 | **-0.010 (-0.020, -0.001)** | 0.12 ± 0.05 | 0.13 ± 0.05 | -0.010 (-0.022, 0.002) |
| Rod bone volume fraction | 0.13 ± 0.03 | 0.13 ± 0.02 | **-0.007 (-0.013, -0.002)** | 0.11 ± 0.02 | 0.11 ± 0.03 | 0.005 (-0.001, 0.011) |
| Plate-rod bone volume ratio | 0.59 ± 0.29 | 0.61 ± 0.29 | -0.056 (-0.126, 0.015) | 1.16 ± 0.64 | 1.28 ± 0.68 | **-0.175 (-0.337, -0.013)** |
| Axial bone volume fraction | 0.09 ± 0.04 | 0.10 ± 0.03 | **-0.010 (-0.019, -0.002)** | 0.13 ± 0.04 | 0.13 ± 0.04 | -0.007 (-0.016, 0.003) |
| Plate tissue fraction | 0.35 ± 0.10 | 0.36 ± 0.10 | -0.023 (-0.047, 0.001) | 0.50 ± 0.12 | 0.53 ± 0.12 | **-0.033 (-0.063, -0.003)** |
| Rod tissue fraction | 0.65 ± 0.10 | 0.64 ± 0.10 | 0.023 (-0.001, 0.047) | 0.50 ± 0.12 | 0.47 ± 0.12 | **0.033 (0.003, 0.063)** |
| Trabecular plate number density (1/mm) | 1.02 ± 0.16 | 1.05 ± 0.14 | **-0.049 (-0.083, -0.015)** | 1.13 ± 0.13 | 1.15 ± 0.11 | -0.022 (-0.051, 0.007) |
| Trabecular rod number density (1/mm) | 1.45 ± 0.10 | 1.47 ± 0.07 | -0.018 (-0.038, 0.003) | 1.36 ± 0.11 | 1.34 ± 0.12 | 0.022 (-0.006, 0.050) |
| Mean trabecular plate thickness (mm) | 0.22 ± 0.01 | 0.22 ± 0.01 | -0.001 (-0.004, 0.002) | 0.24 ± 0.02 | 0.24 ± 0.02 | -0.002 (-0.005, 0.002) |
| Mean trabecular rod thickness (mm) | 0.22 ± 0.01 | 0.22 ± 0.01 | **-0.003 (-0.006, -0.001)** | 0.22 ± 0.01 | 0.22 ± 0.01 | 0.0003 (-0.0026, 0.0032) |
| Mean trabecular plate surface area (mm^2^) | 0.29 ± 0.02 | 0.29 ± 0.02 | **-0.0046 (-0.0089, -0.0002)** | 0.32 ± 0.03 | 0.33 ± 0.03 | -0.006 (-0.013, 0.001) |
| Mean trabecular rod length (mm) | 1.04 ± 0.02 | 1.04 ± 0.02 | 0.002 (-0.004, 0.007) | 1.03 ± 0.02 | 1.04 ± 0.02 | -0.003 (-0.008, 0.002) |
| Plate-plate junction density (1/mm^3^) | 2.36 ± 0.89 | 2.51 ± 0.78 | **-0.244 (-0.429, -0.059)** | 2.61 ± 0.66 | 2.64 ± 0.57 | -0.057 (-0.202, 0.089) |
| Plate-rod junction density (1/mm^3^) | 3.38 ± 1.07 | 3.58 ± 0.89 | **-0.299 (-0.516, -0.082)** | 3.33 ± 0.78 | 3.33 ± 0.72 | -0.009 (-0.186, 0.169) |
| Rod-rod junction density (1/mm^3^) | 2.36 ± 0.58 | 2.43 ± 0.50 | -0.049 (-0.180, 0.082) | 1.84 ± 0.64 | 1.77 ± 0.70 | 0.107 (-0.057, 0.271) |

Standard segmentation. *Differences in least squares means between EDIC participants and control subjects without diabetes obtained from separate generalized estimating equation models adjusted for age, sex, menopause status, weight, height, 25(OH)D ng/mL, osteoporosis medication, and oral glucocorticoids. Boldface indicates significance at p≤0.05.

**Table S2. CLA Parameters among EDIC Participants with T1D and Controls without Diabetes**

|  | **Distal Radius** | | | **Distal Tibia** | | |
| --- | --- | --- | --- | --- | --- | --- |
|  | **T1D**  **(n=183)** | **Controls (n=94)** |  | **T1D**  **(n=183)** | **Controls (n=94)** |  |
| **CLA Parameters** | Mean ± SD | Mean ± SD | Adjusted difference*  (95% CI) | Mean ± SD | Mean ± SD | Adjusted difference*  (95% CI) |
| Endosteal layer |  |  |  |  |  |  |
| Total pore area (mm^2^) | 0.175 ± 0.189 | 0.143 ± 0.116 | 0.016 (-0.018, 0.051) | 1.158 ± 0.682 | 1.148 ± 0.872 | -0.160 (-0.408, 0.087) |
| Total pore number | 4.877 ± 6.871 | 3.756 ± 2.529 | 0.633 (-0.381, 1.647) | 27.007 ± 14.449 | 25.232 ± 15.642 | -1.949 (-6.365, 2.467) |
| Average pore size (mm^2^) | 0.036 ± 0.006 | 0.036 ± 0.006 | -0.0001 (-0.0018, 0.0017) | 0.042 ± 0.005 | 0.044 ± 0.007 | -0.0019 (-0.0040, 0.0003) |
| Midcortical layer |  |  |  |  |  |  |
| Total pore area (mm^2^) | 0.335 ± 0.275 | 0.275 ± 0.244 | 0.025 (-0.041, 0.091) | 2.122 ± 1.176 | 1.965 ± 1.585 | -0.003 (-0.388, 0.383) |
| Total pore number | 7.347 ± 4.868 | 6.358 ± 4.901 | 0.464 (-0.778, 1.705) | 41.637 ± 19.844 | 38.311 ± 26.023 | 0.897 (-5.678, 7.472) |
| Average pore size (mm^2^) | 0.043 ± 0.010 | 0.041 ± 0.007 | 0.001 (-0.001, 0.003) | 0.050 ± 0.010 | 0.049 ± 0.009 | 0.0005 (-0.0028, 0.0037) |
| Periosteal layer |  |  |  |  |  |  |
| Total pore area (mm^2^) | 0.104 ± 0.107 | 0.075 ± 0.078 | 0.009 (-0.013, 0.031) | 1.087 ± 0.928 | 0.841 ± 0.669 | 0.059 (-0.174, 0.291) |
| Total pore number | 2.408 ± 2.209 | 1.851 ± 1.635 | 0.153 (-0.311, 0.616) | 25.828 ± 17.772 | 24.001 ± 21.060 | -1.563 (-7.998, 4.871) |
| Average pore size (mm^2^) | 0.040 ± 0.010 | 0.039 ± 0.010 | 0.0001 (-0.0026, 0.0028) | 0.040 ± 0.007 | 0.036 ± 0.008 | **0.003 (0.001, 0.005)** |
| Entire compartment |  |  |  |  |  |  |
| Total pore area (mm^2^) | 0.614 ± 0.453 | 0.493 ± 0.397 | 0.049 (-0.052, 0.149) | 4.368 ± 2.166 | 3.954 ± 2.713 | -0.201 (-0.927, 0.526) |
| Total pore number | 14.63 ± 10.10 | 11.97 ± 8.35 | 1.450 (-0.610, 3.510) | 94.47 ± 39.67 | 87.55 ± 47.81 | -4.333 (-17.183, 8.518) |
| Average pore size (mm^2^) | 0.040 ± 0.007 | 0.039 ± 0.006 | 0.0004 (-0.0014, 0.0021) | 0.044 ± 0.006 | 0.043 ± 0.006 | 0.001 (-0.001, 0.002) |

Standard segmentation. *Differences in least squares means between EDIC participants and control subjects without diabetes obtained from separate generalized estimating equation models adjusted for age, sex, menopause status, weight, height, 25(OH)D ng/mL, osteoporosis medication, and oral glucocorticoids. Boldface indicates significance at p≤0.05.

**DCCT/EDIC Research Group as of July 1, 2024**

*Study Chairpersons* – D.M. Nathan (chair), R. Gubitosi-Klug (co-chair); *Past*: O. Crofford, B. Zinman; *Deceased*: S. Genuth

*Editor, EDIC Publications* – D.M. Nathan, R. Gubitosi-Klug

**Clinical Centers**

Case Western Reserve University – *Current*: R. Gubitosi-Klug, L. Mayer, J. Wood, G. Greanoff, D. Miller, M. Novak, S. Pendegast, S. Rath, L. Singerman, D. Weiss, H. Zegarra; *Past*: E. Brown, P. Crawford, M. Palmert, P. Pugsley, J. Quin, S. Smith-Brewer; *Deceased*: W. Dahms, S. Genuth, J. McConnell

Weill Cornell Medical College – *Current*: N.S. Gregory, R. Hanna, R. Chan, S. Kiss, A. Orlin, M. Rubin; *Past*: S. Barron, B. Bosco, D. Brillon, S. Chang, A. Dwoskin, M. Heinemann, L. Jovanovic, M.E. Lackaye, T. Lee, B. Levy, V. Reppucci, M. Richardson; *Deceased*: R. Campbell

Henry Ford Health System – *Current*: A. Bhan, J.K. Jones, D. Kruger, P.A. Edwards, S. Mukhashen; *Past*: E. Angus, A. Galprin, M. McLellan, H. Remtema, A. Thomas; *Deceased*: J.D. Carey, F. Whitehouse

International Diabetes Center – *Current*: R. Bergenstal, S. Dunnigan, M. Johnson, A. Carlson, L. Thomas; *Past*: R. Birk, P. Callahan, G. Castle, R. Cuddihy, M. Franz, D. Freking, L. Gill, J. Gott, K. Gunyou, P. Hollander, D. Kendall, J. Laechelt, S. List, G. Matfin, W. Mestrezat, J. Nelson, B. Olson, N. Rude, M. Spencer; *Deceased*: D. Etzwiler, K. Morgan

Joslin Diabetes Center – *Current*: L.P. Aiello, E. Golden, A. Taliaferro, P. Arrigg, J. Cavallerano, R. Cavicchi, M. Elmasry, O. Hamdy, T. Murtha, D. Schlossman, S. Shah, G. Sharuk, P. Silva, P. Silver, M. Stockman, J. Sun, E. Weimann; *Past*: V. Asuquo, R. Beaser, L. Bestourous, O. Ganda, A. Jacobson, R. Kirby, L. Rand, J. Rosenzwieg, H. Wolpert

Massachusetts General Hospital – *Current*: D.M. Nathan, A. Leong, M.E. Larkin, R. Azevedo, R. Bartholomew, T. Bresnahan, K. Chu, J. Heier, C. Shah, N. Thangthaeng; *Past*: E. Anderson, H. Bode, S. Brink, M. Cayford, M. Christofi, C. Cornish, D. Cros, S. Crowell, L. Delahanty, A. deManbey, K. Folino, S. Fritz, C. Gauthier-Kelly, J. Godine, L. Gurry, C. Haggan, K. Hansen, F. Leandre, P. Lou, J. Lynch, K. Martin, C. McKitrick, D. Moore, D. Norman, M. Ong, E. Ryan, C. Stevens, C. Taylor, D. Zimbler

Mayo Clinic – *Current*: A. Vella, K. Osmundson, A. Barkmeier; *Past*: B. French, M. Haymond, J. Mortenson, J. Pach, R. Rizza, L. Schmidt, W.F. Schwenk, R. Woodwick, G. Ziegler, A. Zipse; *Deceased*: R. Colligan, A. Lucas, F.J. Service, B. Zimmerman

Medical University of South Carolina – *Current*: H. Karanchi, L. Spillers, J. Fernandes, K. Hermayer; *Past*: A. Blevins, M. Bracey, S. Caulder, J. Colwell, S. Elsing, A. Farr, S. Kwon, D. Lee, P. Lindsey, M. Lopes-Virella, L. Luttrell, T. Lyons, R. Mayfield, J. Parker, N. Patel, C. Pittman, J. Selby, J. Soule, M. Szpiech, T. Thompson, D. Wood, S. Yacoub-Wasef

Northwestern University – *Current*: A. Wallia, M. Colucci, C. Coventry, M. Gill, A. Lyon, R. Mirza; *Past*: M. El Muayed, D. Adelman, S. Colson, M. Hartmuller, M. Molitch, B. Schaefer

University of California, San Diego – *Current*: S. Mudaliar, G. Lorenzi, O. Kolterman, M. Goldbaum; *Past*: T. Clark, M. Giotta, I. Grant, K. Jones, R. Lyon, M. Prince, R. Reed, M. Swenson; *Deceased*: G. Friedenberg

University of Iowa – *Current*: W.I. Sivitz, B. Vittetoe; *Past*: M. Bayless, C. Fountain, B. Ginsburg, R. Hoffman, J. Kramer, J. MacIndoe, N. Olson, H. Schrott, L. Snetselaar, T. Weingeist, R. Zeitler

University of Maryland – *Current*: R. Miller, P. Newton, S. Johnsonbaugh; *Past*: M. Carney, D. Counts, T. Donner, J. Gordon, M. Hebdon, R. Hemady, B. Jones, A. Kowarski, R. Liss, S. Mendley, D. Ostrowski, M. Patronas, P. Salemi, S. Steidl

University of Michigan – *Current*: W.H. Herman, C.L. Martin, P. Lee,  J. W. Albers, E.L. Feldman S. Kuo; *Past*: N R. Pop-Busui, Burkhart, D.A. Greene, T. Sandford, M.J. Stevens; *Deceased*: J. Floyd

University of Minnesota – *Current*: A. Bantle, J. Bantle, M. Rhodes, D. Koozekanani, S. Montezuma; *Past:* N. Flaherty, F. Goetz, C. Kwong, L. McKenzie, M. Mech, J. Olson, B. Rogness, T. Strand, J. Terry, R. Warhol, N. Wimmergren

University of Missouri – *Current*: D. Hainsworth, S. Hitt, A. Jarvis; *Past:* D. Goldstein; *Deceased*: J. Giangiacomo

University of New Mexico – *Current*: D.S. Schade, A. Korbin, E. Duran-Valdez, R.B. Avery, J.E. Chapin, A. Das, L.H. Ketai; *Past*: M.R. Burge, J.L. Canady, D. Hornbeck, C. Johannes, J. Rich, M.L Schluter

University of Pennsylvania – *Current*: M. Schutta, P.A. Bourne, A. Brucker; *Past*: S. Braunstein, B.J. Maschak-Carey, S. Schwartz; *Deceased*: L. Baker

University of Pittsburgh – *Current*: T. Costacou, F. Toledo, T. Orchard, B.A. Coonrod; *Past*: D. Becker, L. Cimino, B. Doft, D. Finegold, K. Kelly, L. Lobes, D. Rubinstein, N. Silvers, T. Songer, D. Steinberg, L. Steranchak, J.Wesche; *Deceased*: A. Drash

University of South Florida – *Current*: H. Rodriguez, J. O’Brian, Dr Bhaleeya; *Past*: L. Babbione, M.L. Bernal,T.J. DeClue, N. Grove, D. McMillan, A. Morrison, P.R. Pavan, H. Solc, E.A. Tanaka, J. Vaccaro-Kish; *Deceased*: J.I. Malone

University of Tennessee – *Current*: S. Dagogo-Jack, R. Wilson, S. Huddleston, B. Cain; *Past*: M. Bryer-Ash, E. Chaum, A. Iannacone, H. Lambeth, D. Meyer, S. Moser, M.B. Murphy, A. Patel, H. Ricks, S. Schussler, C. Wigley, S. Yoser; *Deceased*: A. Kitabchi

University of Texas Southwestern Medical Center – *Current*: P. Raskin, L. Jordan, B. Shao, YG. He, E. Mendelson, RL. Ufret-Vincenty; *Past*: M. Basco, E. Mendelson; *Deceased*: S. Cercone, S. Strowig

University of Toronto – *Current*: B.A. Perkins, C. M. Falappa, A. Orszag, D. Olegario; *Past*: A. Barnie, D. Daneman, R. Ehrlich, S. Ferguson, A. Gordon, L. Leiter, K. Perlman, S. Rogers, L. Tuason, B. Zinman

University of Washington – *Current*: I. Hirsch, X. Averkiou, L. Van Ottingham, L. Olmos de Koo; *Past*: I.H. de Boer, S. Catton, R. Fahlstrom, J. Kinyoun; *Deceased*: J. Palmer, J. Ginsberg

University of Western Ontario – *Current*: C. McDonald, M. Driscoll, J. Bylsma, T. Sheidow; *Past*: W. Brown, C. Canny, P. Colby, S. Debrabandere, J. Harth, I. Hramiak, M. Jenner, J. Mahon, D. Nicolle, N.W. Rodger, T. Smith ; *Deceased*: J. Dupre

Vanderbilt University – *Current*: K. Niswender, T. Marksbury, T. Adkins, A. Agarwal, C. Lovell; *Past*: S. Feman, J. Lipps Hagan, R. Lorenz, M. May, R. Ramker; *Deceased*: L. Survant

Washington University, St. Louis – *Current*: A. Brown, N.H. White, E. Hoffman; *Past*: L. Levandoski; *Deceased*: I. Boniuk, J. Santiago

Yale University – *Current*: J. Sherr, P. Gatcomb; *Past*: J. Ahern, K. Stoessel, W. Tamborlane

Albert Einstein – *Past*: J. Brown-Friday, J. Crandall, H. Engel, S. Engel, H. Martinez, M. Phillips, M. Reid, H. Shamoon, J. Sheindlin

**Clinical Coordinating Center**

Case Western Reserve University – *Current*: R. Gubitosi-Klug, L. Mayer, K. Farrell, E. Moreau; *Past*: C. Beck, P. Gaston, M. Palmert, J. Quin, R. Trail; *Deceased*: W. Dahms, S. Genuth

**Data Coordinating Center**

George Washington University, The Biostatistics Center – *Current*: J. Lachin, I. Bebu, B. Braffett, M. Bott, B. Burke, L. Diminick, L. El ghormli, X. Gao, D. Kenny, K. Klumpp, M. Lin, V. Trapani; *Past*: K. Anderson, J. Backlund, K. Chan, P. Cleary, A. Determan, L. Dews, S. Ho, W. Hsu, P. McGee, H. Pan, B. Petty, D. Rosenberg, B. Rutledge, W. Sun, S. Villavicencio, N. Younes; *Deceased*: C. Williams

**National Institute of Diabetes and Digestive and Kidney Disease**

National Institute of Diabetes and Digestive and Kidney Disease Program Office – *Current*: E. Leschek, J. Lawrence; *Past*: C. Cowie, C. Siebert

**EDIC Core Central Units**

Central Biochemistry Laboratory (University of Minnesota) – *Current*: M. Steffes, A. Karger, J. Seegmiller, V. Arends; *Past*: J. Bucksa, B. Chavers, A. Killeen, M. Nowicki, A. Saenger

Central ECG Reading Unit (Wake Forest School of Medicine) – Current: E.Z. Soliman, L. Keasler, Y. Li, S. Moldibi, S Belton, I. Karabayir, K. Calloway; Past: Y. Pokharel, R. Prineas, C. Campbell, M. Barr, T. Taylor, Z.M. Zhang, S. Hensley, J. Hu

Central Ophthalmologic Reading Unit (University of Wisconsin) – *Current*: B. Blodi, R. Domalpally, E. Showers; *Past*: Danis, D. Lawrence, H. Wabers, M. Burger, M. Davis, J. Dingledine, V. Gama, S. Gangaputra, L. Hubbard, S. Neill, R. Sussman

Central Neuropsychological Reading Unit (NYU Long Island School of Medicine, University of Pittsburgh) – *Current*: A. Jacobson, C. Ryan, N. Chaytor, D. Saporito; *Past*: B. Burzuk, E. Cupelli, M. Geckle, D. Sandstrom, F. Thoma, T. Williams, T. Woodfill
